# Evaluation of recent changes in genetic variability in Thoroughbred horses based on microsatellite markers parentage panel in Korea

**DOI:** 10.5713/ab.21.0272

**Published:** 2021-08-25

**Authors:** Chul Song Park, Sun Young Lee, Gil Jae Cho

**Affiliations:** 1 College of Veterinary Medicine and Institute of Equine Medicine, Kyungpook National University, Daegu 41566, Korea; 2 Racing Laboratory, Korea Racing Association, Gwacheon 13822, Korea

**Keywords:** Individual Identification, Microsatellite Marker, Parentage Verification, South Korea, Thoroughbred Horse

## Abstract

**Objective:**

In this study, we aimed to investigate the recent changes such as allele frequencies and total probability of exclusion (PE) in Thoroughbred horses in Korea using short tandem repeat (STR) parentage panels between 2006 and 2016.

**Methods:**

The genotype was provided for 5,988 horse samples with 15 microsatellite markers (AHT4, AHT5, ASB2, ASB17, ASB23, CA425, HMS1, HMS2, HMS3, HMS6, HMS7, HTG4, HTG10, LEX3 and VHL20).

**Results:**

In our study, the observed number of alleles per locus ranged from 3 (HMS1) to 9 (ASB17) in 2006 and 4 (HMS1) to 9 (ASB2) in 2016, with a mean value of 6.28 and 6.40, respectively. Of the 15 markers, HMS2, HTG4, and CA425 loci had relatively low polymorphism information content (<0.5000) in the Thoroughbred population. Mean levels of genetic variation in 2006 and 2016 were observed heterozygosity (H_O_) = 0.708, and expected heterozygosity (H_E_) = 0.685, as well as and H_O_ = 0.699 and H_E_ = 0.682, respectively. The PE was calculated for each group based on the allele frequencies of 14 or 15 STRs. The 2006 survey analyzed that PE was 0.9998, but it increased to 0.9999 in 2016 after the HMS2 marker was added in 2011. The current STR panel is still a powerful tool for parentage verification that contributes to the maintenance of integrity in the Thoroughbred population.

**Conclusion:**

The current STR panel is still a powerful tool for parentage verification that contributes to the maintenance of integrity in the Thoroughbred horses. However, continuous monitoring genetic variability is necessary.

## INTRODUCTION

The first volume of Thoroughbred Stud Book was published in England in 1791 [1], and the first volume was published in Korea in 1998. Domestic foals can be listed in the Korean Stud Book (KSB) through appearance screening and paternity verification. In Korea, the paternity of Thoroughbred horses was tested via blood type until the late 1990s but was changed to microsatellite DNA analysis in 2002 [2].

Microsatellite polymorphism, i.e., short tandem repeat (STR) in a horse, was first reported by Ellegren et al [3] and Marklund et al [4]. Microsatellites have been used for genetic diversity assessment, genetic maps constructions, quantitative trait loci mapping and parentage testing [5,6]. The STR became a valuable tool for horse parentage verification and pedigree registration [7]. In 1998, at the Equine Genetic and Thoroughbred parentage Testing workshop hosted by the International Society for Animal Genetics (ISAG), an international panel with nine STRs for horse DNA typing was created, in 2011, three STRs were added, with 12 STRs currently recommended by ISAG. The International Stud Book Committee (ISBC) recommends using these 12 STRs for registration of Thoroughbred horses. It also requires that the total probability of exclusion (PE) value of parentage testing for Thoroughbred horses exceeds 0.9995 [8].

In Korea, Thoroughbred horse parentage testing started in 2002 using 14 STRs, including the nine (AHT4, AHT5, ASB2, HMS3, HMS6, HMS7, HTG4, HTG10, VHL20) STRs recommended by ISAG. In total, 15 STRs, including 12 (9 STRs plus ASB17, ASB23, HMS2) recommended by ISAG, have been routinely used since 2013.

At present, in South Korea, ~1,000 donkeys and ~27,000 domestic horses exist, including 12,000 Thoroughbreds, 1,000 individuals from other horse breeds (e.g., Warmblood, Quarter horse), and 14,000 native horses (the Jeju Halla horse), of which ~5,000 Jeju horses which was designated as natural monument No. 347 by the government [9].

In Korea, ≥1,500 Thoroughbred foals are born annually. In order for domestic-born Thoroughbred horses to be used as racehorses, they must be identified through appearance tests or genetic tests to be listed in the KSB. In addition, the horse's microsatellite DNA marker used in Korea was recognized for the registration of Thoroughbred horses from ISAG.

Stallions in Korea, have been replaced a lot so far; hence genetic diversity is also presumed to have changed. Therefore, in this study, we investigated the recent changes such as allele frequencies and PE in Thoroughbred horses in Korea using STR parentage panels between 2006 and 2016.

## MATERIALS AND METHODS

### Ethical statement

This study was a routine test for the registration of Thoroughbred horses in Korea and not approved by Research Ethical Committee, but sampling of this study was performed according to the guidelines of the Animal Ethics Committee of Korea Racing Authority in Korea for the care and use of experimental animals.

### Sample collection and DNA extraction

A total of 5,988 Thoroughbred horses were examined. Genomic DNA was extracted from hair root samples using a MagExtractor System MFX-6100 (Toyobo, Osaka, Japan) according to the manufacturer's instructions [8].

### Microsatellite markers and analysis

**Figure 1:**
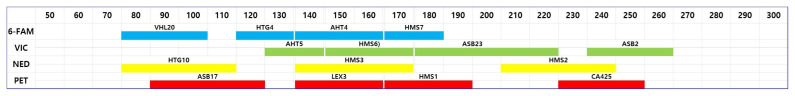
Allelic range of each marker including fluorescent dye used in this study.

A total of 15 microsatellite loci (AHT4, AHT5, ASB2, ASB17, ASB23, CA425, HMS1, HMS2, HMS3, HMS6, HMS7, HTG4, HTG10, LEX3, and VHL20) were used for the analysis of Thoroughbred horses. Detailed characteristics of 15 STRs is as shown in Table 1. The fluorescent dye attached to the markers used in this study and the marker sizes (DNA base pair) shown in Figure 1. Polymerase chain reaction (PCR) was performed according to the manufacturer’s protocols (Applied Biosystems, Waltham, MA, USA). Of the 15 markers, HTG10 was examined by a single PCR.

The PCR was performed with a total volume of 15 μL of the following mixture: 40 ng of genomic DNA, 4.0 μL of each primer, 1.25 mM of dNTPs, 2.5 μL of 10× reaction buffer, and 5 U of *Taq* polymerase (Applied Biosystems, USA). For the single PCR, template DNA 2 μL, 3 pmol forward and reverse primers 2 μL each, and sterile distilled water 6.5 μL were mixed with PCR Premix buffer (Qiagen, Hilden, Germany), and adjusted to 25 μL.

Multiplex PCR amplification was as follows: initial denaturation for 10 min at 95°C, followed by 30 cycles at 95°C for 30 s, 60°C for 30 s and 72°C for 1 min. An extension step at 72°C for 60 min was added after the final cycle [10]. The single PCR was as follows: initial denaturation for 3 min at 95°C, followed by 30 cycles at 95°C for 45 s, 56°C for 30 s and 72°C for 1 min. An extension step at 72°C for 60 min was added after the final cycle.

The PCR was performed using a GeneAmp PCR System 9700 (Applied Biosystems, USA). The PCR products were electrophoresed and then analyzed using an ABI 3130 xl Genetic Analyzer (Applied Biosystems, USA). The genotype of each of the 15 STRs was determined using Genotyper/Genemapper software Ver.3.7 (Applied Biosystems, USA). Then, with the peak row data, the size of allelic genes (base pair) for each marker was determined based on the result of horse comparison test for STR conducted by ISAG.

### Statistical analysis

Allelic frequencies, the number of alleles per locus were estimated by direct counting from observed genotype, and the observed heterozygosity (H_O_), expected heterozygosity (H_E_), polymorphism information content (PIC), and PE were computed using the Cervus ver. 3.0.3 [11].

## RESULTS

### Allele frequencies and expected heterozygosities

In our study, 15 microsatellites were used to identify the genetic diversity of Thoroughbred horses. Allele frequency at each locus is shown in Table 2. The observed number of alleles per locus ranged from 3 (HMS1) to 9 (ASB17) in 2006 and 4 (HMS1) to 9 (ASB2) in 2016, with a mean value of 6.28 and 6.40, respectively. Of the 15 markers, HMS2, HTG4, and CA425 loci had relatively low PIC (<0.5000) in Thoroughbred horses. Mean levels of genetic variation in 2006 and 2016 were H_O_ = 0.708 and H_E_ = 0.685 as well as H_O_ = 0.699 and H_E_ = 0.682, respectively (Tables 3 and 4).

### Exclusion probability

The total PE was calculated for each group based on the allele frequencies of 14 or 15 STRs. The 2006 survey analyzed that PE was 0.9998, but it increased to 0.9999 in 2016 after the HMS2 marker was introduced in 2011.

## DISCUSSION

Several microsatellite markers have been isolated from the horse genome [12], and the microsatellites showed multiple alleles and high heterozygosity among European horse breeds such as Thoroughbred horse. Several types of microsatellites are informative because of their high polymorphism, and they are useful in paternity testing of animals. In cattle, pig, horse, dog, and other populations, pedigree control has been performed on a routine basis in most countries [12–18]. These controls rely on microsatellite typing standardized through regular comparison tests with aid from the ISAG.

Blood typing tests of horses require fresh whole blood and include the costs of sampling by veterinarians, and transportation due to rapid transportation; the entire test process is manually conducted by humans. The results were also slightly inaccurate, with 97% to 99.5% in probability of parentage verification. However, since 2002, microsatellite DNA typing was introduced and used for the paternity assessment of horses, most of these deficiencies in existing blood typing tests have been resolved. In Korea, a comparative analysis of the number of alleles, frequency of alleles, Ho, He, and PIC with microsatellite markers used in 2006 and 2016 showed that many stallions and mares had been replaced, but genetic diversity did not change significantly because of the small numbers of mares and stallions raised in the country. The total PE of the STR panel has changed significantly. This trend continues after adding HMS2 in 2011. Nevertheless, the total PE values for 14 or 15 STRs in Thoroughbred horses in Korea have been greater than the ISBC recommended values (>0.9995) over the past decade.

In Korea, 15 STR markers are used to conduct parentage verification for approximately 1,500 Thoroughbred horses in a year. Parentage verification is determined using Mendel's genetic law. If a discrepancy occurs in one marker after the test judgment, it is assumed to be a mutation and additionally examined with three to five TKY marker; if two or more markers deviate from Mendel's genetic law, it is finally determined that they are not paternity. In general, approximately 10 to 12 heads (0.7% to 0.8%) per year are not paternity.

On the basis of the PIC of each marker, the validity and reliability of the marker can be estimated. If the PIC value is ≥0.5000, the reliability of the marker is valid for pedigree analysis. If it is ≥0.7000, it has universal validity for analysis and can get a highly reliable result can be obtained.

It is currently under discussion and research to introduce single nucleotide polymorphism (SNP) instead of microsatellite DNA markers for parentage verification and individual identification of Thoroughbred horses. However, the results of this study are expected to be valid until the introduction of the SNP in the Thoroughbred population in Korea.

## Figures and Tables

**Table 1:** Characteristics of the 15 microsatellite loci used in this study

Loci	Primer sequence (5′ → 3′)
VHL20	CAAGTCCTCTTACTTGAAGACTAG, AACTCAGGGAGAATCTTCCTCAG
HTG4	CTATCTCAGTCTTGATTGCAGGAC, CTCCCTCCCTCCCTCTGTTCTC
AHT4	AACCGCCTGAGCAAGGAAGT, GCTCCCAGAGAGTTTACCCT
HMS7	CAGGAAACTCATGTTGATACCATC, TGTTGTTGAAACATACCTTGACTGT
AHT5	ACGGACACATCCCTGCCTGC, GCAGGCTAAGGGGGCTCAGC
HMS6	GAAGCTGCCAGTATTCAACCATTG, CTCCATCTTGTGAAGTGTAACTCA
ASB2	CCACTAAGTGTCGTTTCAGAAGG, CACAACTGAGTTCTCTGATAGG
HTG10	CAATTCCCGCCCCACCCCCGGCA, TTTTTATTCTGATCTGTCACATTT
HMS3	CCAACTCTTTGTCACATAACAAGA, CCATCCTCACTTTTTCACTTTGTT
HMS2	CTTGCAGTCGAATGTGTATTAAAT, ACGGTGGCAACTGCCAAGGAAG
ASB17	GAGGGCGGTACCTTTGTACC, ACCAGTCAGGATCTCCACCG
CA425	AGCTGCCTCGTTAATTCA, CTCATGTCCGCTTGTCTC
ASB23	GCAAGGATGAAGAGGGCAGC, CTGGTGGGTTAGATGAGAAGTC
HMS1	CATCACTCTTCATGTCTGCTTGG, TTGACATAAATGCTTATCCTATGGC
LEX3	ACACTCTAACCAGTGCTGAGACT, GAAGGAAAAAAAGGAGGAAGAC

**Table 2:** Allelic frequencies calculated from 5,988 Thoroughbred horses

Markers	Years	Alleles and frequencies DNA type															

		B^1)^	F	G	H	I	J	K	L	M	N	O	P	Q	R	S	U
AHT4	2006	-	-	-	0.2105	-	0.2241	0.1732	0.0004	-	-	0.3918	-	-	-	-	-
	2016	-	-	-	0.1329	-	0.2354	0.2104	-	-	0.0004	0.4208	0.0001	-	-	-	-
AHT5	2006	-	-	-	-	-	0.1393	0.4911	-	0.2136	0.1233	0.0327	-	-	-	-	-
	2016	-	-	-	-	-	0.2039	0.4678	0.0004	0.2112	0.0965	0.0201	-	-	-	-	-
ASB2	2006	0.0307	-	-	-	-	-	0.1304	-	0.1331	0.1366	0.1163	0.0117	0.2634	0.1778	-	-
	2016	0.0245	-	-	-	-	-	0.0893	0.0001	0.2063	0.1501	0.0725	0.0068	0.264	0.1864	-	-
HMS1	2006	-	-	-	-	0.1739	0.4553		-	0.3708	-	-	-	-	-	-	-
	2016	-	-	-	-	0.1856	0.4124	0.0001	-	0.4019	-	-	-	-	-	-	-
HMS2	2006	-	-	-	-	-	-	-	-	-	-	-	-	-	-	-	-
	2016	-	-	-	0.0198	-	0.0557	0.1375	0.7407	0.0464	-	-	-	-	-	-	-
HMS3	2006	-	-	-	-	0.5475	-	0.0004	-	0.1451	0.0393	0.1089	0.1467	-	0.0121	-	-
	2016	-	-	-	-	0.5516	-	-	-	0.1274	0.0116	0.1383	0.1622	-	0.0089	-	-
HMS6	2006	-	-	-	-	-	-	0.1323	0.0276	0.2926	-	0.014	0.5319	-	0.0016	-	-
	2016	-	-	-	-	-	-	0.1321	0.0353	0.3182	-	0.0023	0.5118	0.0003	-	-	-
HMS7	2006	-	-	-	-	-	0.0864	0.0012	0.1412	0.2385	0.2023	0.3304	-	-	-	-	-
	2016	-	-	-	-	-	0.0854	-	0.1511	0.3065	0.2424	0.2145	-	-	0.0001	-	-
HTG4	2006	-	-	-	-	-	-	0.535	0.0012	0.4058	0.0249		0.0331	-	-	-	-
	2016	-	-	-	-	-	-	0.5659	0.0001	0.3764	0.0346	0.0001	0.0230	-	-	-	-
HTG10	2006	-	-	-	-	0.2782	-	0.1245	0.1669	0.1833		0.0934	-	0.0008	0.1518	0.0012	-
	2016	-	-	-	-	0.2826	0.0001	0.1173	0.2317	0.1863		0.0879	-	-	0.0001	0.0007	-
VHL20	2006	-	-	-	-	0.2805	-	-	0.2307	0.3463	0.1374	0.0051	-	-	-	-	-
	2016	-	-	-	-	0.2956	-	0.0001	0.2251	0.292	0.1854	0.0018	-	-	-	-	-
ASB17	2006	-	-	0.3261	0.0016	-	-	-	-	0.0315	0.2533	0.2171	0.0008	0.0008	0.1685	0.0004	-
	2016	-	-	0.3756	0.0013	-	-	-	-	0.0351	0.1869	0.2314	-	0.0006	0.1690	-	-
ASB23	2006	-	-	-	-	0.0704	0.2817	0.2424	0.1782	-	-	-	-	-	-	0.2058	0.0214
	2016	-	-	-	-	0.0949	0.3375	0.2075	0.1706	-	-	-	-	-	-	0.1626	0.0303
LEX3	2006	-	0.0039	-	0.2665	-	0.0004	-	0.0198	0.1362	0.1171	0.0529	0.4031	-	-	-	-
	2016	-	0.0045	-	0.2787	-	-	-	0.0237	0.0936	0.086	0.0703	0.2352	-	-	-	-
CA425	2006	-	-	-	-	0.0323	0.1786	0.0019	0.0132	0.0268	0.6342	0.1128	-	-	-	-	-
	2016	-	-	-	-	0.0139	0.1551	0.0129	0.0116	0.0134	0.6444	0.1485	0.0002	-	-	-	-

1) Alphabetical allele code for all loci are identical to the assignment from International Society for Animal Genetics.

**Table 3:** Number of alleles, heterozygosity, PIC value and PE of 15 microsatellite markers in 5,988 Thoroughbred horses

Markers	Years	No. of alleles	H_O_	H_E_	PIC	PE
AHT4	2006	5	0.781	0.722	0.674	0.474
	2016	6	0.710	0.706	0.656	0.455
AHT5	2006	5	0.685	0.678	0.634	0.443
	2016	6	0.689	0.685	0.683	0.442
ASB2	2006	8	0.852	0.831	0.809	0.663
	2016	9	0.817	0.817	0.791	0.636
HMS1	2006	3	0.626	0.625	0.547	0.334
	2016	4	0.616	0.634	0.556	0.341
HMS2	2006					
	2016	5	0.420	0.427	0.400	0.243
HMS3	2006	7	0.645	0.644	0.608	0.424
	2016	6	0.607	0.634	0.597	0.405
HMS6	2006	6	0.618	0.613	0.551	0.352
	2016	6	0.629	0.618	0.551	0.349
HMS7	2006	6	0.767	0.766	0.728	0.545
	2016	6	0.777	0.771	0.734	0.551
HTG4	2006	5	0.559	0.548	0.451	0.257
	2016	6	0.537	0.536	0.444	0.252
HTG10	2006	8	0.804	0.814	0.788	0.630
	2016	8	0.808	0.802	0.773	0.609
VHL20	2006	5	0.736	0.730	0.680	0.479
	2016	6	0.737	0.742	0.695	0.495
ASB17	2006	9	0.762	0.753	0.710	0.520
	2016	7	0.752	0.741	0.698	0.507
ASB23	2006	6	0.779	0.783	0.748	0.571
	2016	6	0.784	0.779	0.747	0.573
LEX3	2006	8	0.756	0.731	0.691	0.505
	2016	7	0.850	0.802	0.774	0.612
CA425	2006	7	0.553	0.551	0.513	0.332
	2016	8	0.544	0.538	0.498	0.317

H_O_, observed heterozygosity; H_E_, expected heterozygosity; PIC, polymorphism information content; PE, probability of exclusion.

**Table 4:** Allele diversity (the mean number alleles per locus), mean levels of genetic variation in 2006 and 2016 in Thoroughbred horses

Years	Sample size	Allele diversity	H_O_	H_E_	PIC	PE
2006	1,285	6.28	0.708	0.699	0.652	0.9998
2016	4,703	6.40	0.685	0.682	0.639	0.9999

H_O_, observed heterozygosity; H_E_, expected heterozygosity; PIC, polymorphism information content; PE, total probability of exclusion.
